# Gravity-induced coronal plane joint moments in adolescent idiopathic scoliosis

**DOI:** 10.1186/s13013-015-0060-9

**Published:** 2015-12-17

**Authors:** Bethany E. Keenan, Graeme J. Pettet, Maree T. Izatt, Geoffrey N. Askin, Robert D. Labrom, Mark J. Pearcy, Clayton Adam

**Affiliations:** Paediatric Spine Research Group, Institute of Health and Biomedical Innovation, Queensland University of Technology and Mater Health Services, Brisbane, 4101 Queensland Australia; Institute of Health and Biomedical Innovation, Queensland University of Technology, Brisbane, QLD Australia

**Keywords:** Adolescent idiopathic scoliosis (AIS), Computed tomography (CT), Gravity, Imaging, Instantaneous centre of rotation (ICR), Joint moments, Scoliosis, Scoliosis progression

## Abstract

**Background:**

Adolescent Idiopathic Scoliosis is the most common type of spinal deformity, and whilst the isk of progression appears to be biomechanically mediated (larger deformities are more likely to progress), the detailed biomechanical mechanisms driving progression are not well understood. Gravitational forces in the upright position are the primary sustained loads experienced by the spine. In scoliosis they are asymmetrical, generating moments about the spinal joints which may promote asymmetrical growth and deformity progression. Using 3D imaging modalities to estimate segmental torso masses allows the gravitational loading on the scoliotic spine to be determined. The resulting distribution of joint moments aids understanding of the mechanics of scoliosis progression.

**Methods:**

Existing low-dose CT scans were used to estimate torso segment masses and joint moments for 20 female scoliosis patients. Intervertebral joint moments at each vertebral level were found by summing the moments of each of the torso segment masses above the required joint.

**Results:**

The patients’ mean age was 15.3 years (SD 2.3; range 11.9–22.3 years); mean thoracic major Cobb angle 52^°^ (SD 5.9^°^; range 42–63^°^) and mean weight 57.5 kg (SD 11.5 kg; range 41–84.7 kg). Joint moments of up to 7 Nm were estimated at the apical level. No significant correlation was found between the patients’ major Cobb angles and apical joint moments.

**Conclusions:**

Patients with larger Cobb angles do not necessarily have higher joint moments, and curve shape is an important determinant of joint moment distribution. These findings may help to explain the variations in progression between individual patients. This study suggests that substantial corrective forces are required of either internal instrumentation or orthoses to effectively counter the gravity-induced moments acting to deform the spinal joints of idiopathic scoliosis patients.

**Electronic supplementary material:**

The online version of this article (doi:10.1186/s13013-015-0060-9) contains supplementary material, which is available to authorized users.

## Background

Adolescent Idiopathic Scoliosis (AIS) is a three-dimensional spinal deformity whose aetiology remains unclear [1–4]. Whilst the initial deformity may be due to a complex interplay of biomechanical, biochemical, and/or genetic factors, as well as growth asymmetries originating in the sagittal plane, it is widely accepted that scoliosis progression is predominantly a biomechanical process, whereby the spine undergoes asymmetric loading and alteration of vertebral growth in a *“vicious cycle”* [5, 6].

Supine imaging modalities such as Computed Tomography (CT) and Magnetic Resonance Imaging (MRI) have made possible 3D reconstructions of the spine, which allow detailed measurements of spinal anatomy not possible with standard radiographs. Segmental (vertebral level-by-level) torso masses^1^ can be calculated from these 3D reconstructions and used to determine the gravity-induced joint moments^2^ acting on the scoliotic spine. In the healthy, non-scoliotic spine there are negligible joint moments acting in the coronal plane, whereas, once a small lateral curvature presents, the weight of the torso segments superior to that curve generate a lateral bending moment that can potentially exacerbate the deformity during subsequent growth. For a patient with mild scoliosis, the moment created has been estimated to be in the order of 0.5 Nm [7]. However, it is not yet known whether there is a threshold beyond which joint moments drive deformity progression.

Previous studies suggest that gravitational forces in the standing position play an important role in scoliosis progression. Adam et al. (2008) reported that gravity-induced axial rotation torques may modulate intravertebral rotation in progressive idiopathic scoliosis (as gravitational forces acting on a curved spinal column generate torque about the column axis).

Torques as high as 7.5 Nm were found acting on scoliotic spines in the standing position, but further investigation of this area is required [8]. Spinal loading asymmetry in the lumbar spine with regard to muscle activation has also been extensively reviewed by Stokes [9–11]. However, to the best of our knowledge, there have not been any previous analyses of gravity-induced coronal plane joint moments in the thoracic and lumbar spines of AIS patients.

Given that joint moments in the transverse plane have previously been estimated by Adam et al (2008), the plane of primary interest in the current study is the coronal plane. This is also the plane in which routine scoliosis assessment is performed clinically through the use of standing postero-anterior plane radiographs. Whilst joint moments are also induced in the sagittal plane, the spine is adapted to resist these, since they are present as a consequence of the natural thoracic kyphosis and lumbar lordosis which is present in scoliotic and healthy spines alike.

The aims of the present study were: (i) to estimate torso segment masses in the thoracic and lumbar spines of AIS patients, using a series of existing low-dose supine CT scans, (ii) to calculate the resulting gravity-induced coronal plane joint moments in the adolescent scoliotic spine; and (iii) to assess the relationship between coronal plane joint moments and the severity of the deformity.

## Methods

### Patient cohort and ethical consideration

Existing low-dose supine CT scans taken between November 2002 and January 2008 for a group of female AIS patients were used retrospectively to determine the coronal plane joint moments acting on the thoracic scoliotic spine. All patients had right-sided thoracic curves with a Cobb angle greater than 40°.

The curve type was based on the Lenke classification system [12], and all patients were categorised as Lenke Type 1 (i.e. they had major thoracic curves). The Risser sign was used to categorise the skeletal maturity of each patient [13].

A single low-dose CT scan was part of the pre-operative clinical assessment process at the time, for patients scheduled to undergo thoracoscopic anterior spinal fusion to assist with safer screw sizing and positioning [14]. Ethical and institutional governance approvals were gained prior to commencement of the study.

### CT data evaluation

Three different CT scanners were used over the 6 year period of the CT scan acquisition: (i) a 64 slice GE Lightspeed Plus (GE Healthcare, Chalfont St. Giles, UK); (ii) a 64-slice Philips Brilliance (Philips Healthcare, Andover, USA); and (iii) a 64 slice GE Lightspeed VCT (GE Healthcare, Chalfont St. Giles, UK). Dose reports were commissioned for all three scanners, and the highest estimated radiation dose of 3.0 mSv occurred with the oldest scanner (GE Lightspeed Plus), with uncertainties due to the dose model in the order of 20 % [15].

By comparison, the combined dose for a postero-anterior (PA) and lateral standing radiograph is in the order of 1.0 mSv, and the annual background radiation dose in Queensland, Australia is approximately 2.0–2.4 mSv [15, 16]. Estimated doses for the newer 64 slice scanners were substantially lower (in the order of 2 mSv). Subjects were in a supine position with the upper limbs positioned over the head during CT scanning. Scan coverage was from C7 to S1.

**Fig. 1:**
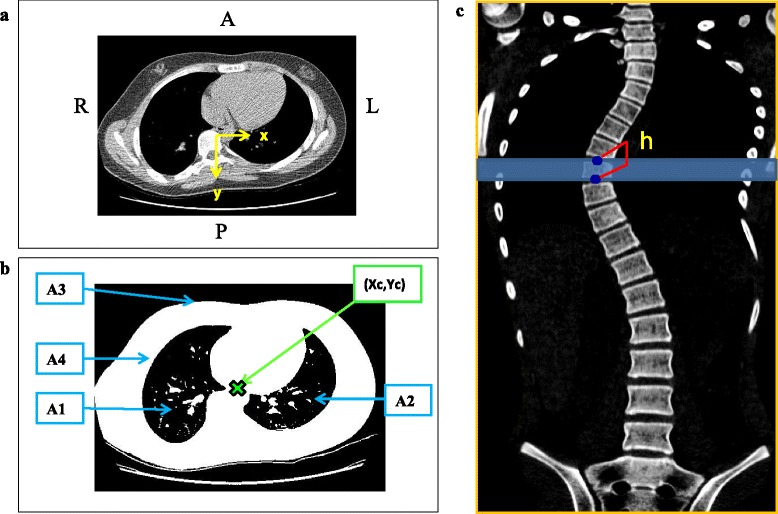
**a** Axial slice through the T8 vertebra showing x and y co-ordinate axes relative to the centroid of T1 **b** Thresholded axial slice at the same level - where A4 is the area of the torso, minus that of the lungs A1 (right lung) and A2 (left lung), A3 is the area of the total slice and (Xc,Yc) denotes the centroid of the cross-section. **c** Torso segment thickness (h) calculated from the reconstructed coronal image, where the segment height was taken from the midpoint of the superior vertebral endplate of each vertebra to the midpoint of the superior vertebral endplate of the level below including the IV disc. The distance between these two points was measured using the ImageJ ‘Segmented Line’ measuring tool

The CT scan in-plane resolution and slice thickness/spacing varied slightly over the course of the study. All raw scans were 16 bit axial stacks with 512 × 512 pixels in each slice of the stack. Pixel spacing in the (axial) plane varied between 1.7 and 1.8 pixels/mm with a slice thickness between 2.0 and 3.0 mm and slice spacing between 1.0 and 1.25 mm. Since the re-sliced coronal plane images (Fig. 1) derive their resolution from the original CT dataset, the pixel spacing in the plane (on the re-sliced stack) varied from 1.7–1.8 pixels/mm in both the lateral (left-right) and anterior-posterior directions, and 0.8–1.0 pixels/mm in the longitudinal (inferior-superior) direction.

The image processing software, ImageJ (v. 1.45, National Institutes of Health, USA) was used to create re-sliced coronal plane images from the axial CT slices and reconstruct vertebral level-by-level torso segments. The scoliotic vertebral level segmental parameters of height, volume, area and mass were measured, to enable estimation of the coronal plane joint moments acting at each level. All analyses were completed by a single observer.

### Estimating vertebral level torso segment masses

A single axial CT slice located at the mid-height of each thoracic and lumbar vertebra was selected and used to determine the axial plane location of the torso segment centroid for that vertebral level as described below.

ImageJ’s default thresholding method based on the IsoData algorithm described by Ridler and Calvard [17] was then used on the axial slice through the centre of each vertebral body to distinguish the external and lung airspaces from the trunk tissues (Fig. 1). The centroid co-ordinates of this thresholded slice (Xc, Yc) in the scanner bed coordinate system were then found using the First Moment of Area equation (Equation 1 and 2).

1 $$ Xc = \frac{(A3X3) - (A1X1) - (A2X2)}{A4} $$

2 $$ Yc = \frac{(A3Y3) - (A1Y1) - (A2Y2)}{A4} $$

Note: *A*4=*A*3-*A*1-*A*2

A3 is the total area of the axial slice enclosed by the skin boundary, A1 is the area of the right lung and A2 is area of the left lung. The centroid co-ordinates of the whole slice are defined as (X3, Y3) with the centroid location of the right and left lungs denoted as (X1, Y1) and (*X*2, Y2) respectively.

The ‘z-project’ function in ImageJ (standard deviation projection type) was used to create a pseudo-coronal plane radiograph to allow the whole thoracic and lumbar spine to be viewed on a single image.

In order to project a clear single image of the thoracic and lumbar spine (without the ribs), the start and end slices for the z-project function were selected at the anterior and posterior edges of the vertebral body. The thickness of each torso segment was measured using techniques described by Keenan et al. [18] where the segment height (or thickness) was taken from the midpoint of the superior vertebral endplate of each vertebra to the midpoint of the superior vertebral endplate of the level below including the IV disc. The volume, V, was then calculated by multiplying the area of the central slice (A4) in the axial plane by the thickness (h) of the vertebral body segment (Fig. 1) corresponding to the vertebra and disc in question.

A single density, ρ, of 1040 kg/m3 [18, 19] was used to estimate the torso segment masses, M, corresponding to each vertebral level (Equation 3).

3 $$ M=\rho \times V $$

**Fig. 2:**
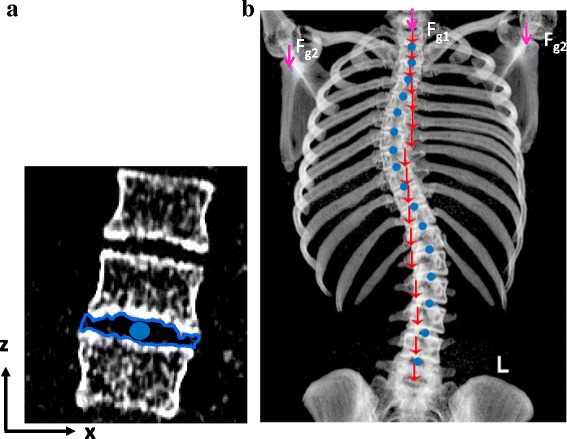
Constructions to calculate segmental moments: **a** using ImageJ polygon tool to trace the outline of a disc space for calculation of the disc’s coronal centroid for the joint’s ICR location (blue dot); **b** coronal reformatted image of the entire thoracic and lumbar spine with overlaid plot of the torso segment weight vectors (indicated by red arrows), the estimated head and upper limb weight vectors (pink arrows) and the ICRs located at the centroids of the IVDs (blue dots)

The torso segment masses were then multiplied by 9.81 m/s^2^ to give torso segment weight vectors. These were plotted at the centroid co-ordinate positions on the patients’ antero-posterior (AP) view images (Fig. 2). Note that although the torso segment weights were estimated based on CT scans performed in the supine position, the resulting gravity vectors were oriented perpendicular to the torso cross-section slices, in order to estimate joint moments in a simulated standing position.

### Anthropometric data

As the CT scans only included the thoracic and lumbar spine and glenohumeral joint, the weight of other body segments above the apex (i.e. the head, neck, arms and hands) were estimated using anthropometric data [20]. Equations 4 and 5 were then used to determine patient specific values for the mass of the head + neck, and for each arm + hand.

4 $$ 8.1\% \times patient\  body\  weight\ (kg) = mass\  of\  head+ neck $$

5 $$ 5.6\% \times patient\  body\  weight\ (kg) = mass\  of\  arm+ hand $$

where the head + neck weight vector was located at the centroid of the T1 superior endplate and the arm + hand weight vector was positioned on the glenohumeral joint (Fig. 2(b)). It is important to note however, that the present literature regarding body segment parameters is limited, particularly for female subjects or adolescents. As a result, the anthropometric body segment percentage values (reported by Winter) are based on measurements of eight male cadavers aged 61–83 years. Whilst we note that this introduces a limitation to the study, AIS patients tend to be leaner and have significantly lower body mass index (BMI) compared to healthy age-matched controls; and therefore are likely to have segment values as a percentage of body mass closer to those of elderly adults [21, 22].

### Locating the Instantaneous Centre of Rotation (ICR)

The next step was to estimate the Instantaneous Centre of Rotation (ICR) location at each intervertebral joint, about which the joint moments would be calculated. Since there is an absence of ICR measurements for lateral bending in scoliosis patients, the ICR was assumed to lie at the centroid of a coronal plane projection of the intervertebral disc.

The ImageJ ‘Polygon’ tool was used to trace the outline of each intervertebral disc (as shown by the blue lines in Fig. 2(a)). Note that the image has been thresholded in this Figure to only display bone, hence the disc is not visible. Once the boundaries of the intervertebral disc had been drawn, the ImageJ ‘Centroid Measurement’ tool was used to determine the centre of this region, which in turn allowed the ICRs to be located in the geometric centres of the discs in the pseudo-coronal plane projection of the patient’s spine. Figure 2(b) shows a plot of the torso segment weight vectors (red arrows) together with the assumed ICRs located in the centre of each disc (blue dots). The lengths of the red arrows are not proportional to the weight of the torso segment, but simply illustrate the location of the vectors. In addition, the pink arrows located on the glenohumeral joint and the centroid of the T_1_ superior endplate represent the estimated torso segment weight vectors of the arm + hands (F_g2_) and head + neck (F_g1_). Note that the most caudal joint moment calculation was performed at the L4/L5 intervertebral joint because of the lack of clarity of the lumbo-sacral joint in a number of the CT scans.

### Calculating gravity-induced coronal plane joint moments

The torso segment weight vectors, together with the assumed ICR locations allowed calculation of the coronal plane joint moments induced by simulated gravitational loading. Intervertebral joint moments, *JM*, at each vertebral level were found by summing the moment of each of the torso segment weight vectors (including the head, neck and arms) about the ICR of the joint in question (Force × perpendicular distance to the ICR).

For example, for the moment acting about the ICR of the T3/T4 joint, the moment contributions from the head and neck, left arm, right arm and T1, T2 and T3 segments were summed to obtain a value for the T3/T4 joint moment.

As the torso segment weight vectors were applied parallel to the z-axis of the CT scanner, it is important to consider the position of each patient on the scanner bed to identify any misalignment of patients during their supine scan. This was assessed by comparing the standing radiograph coronal plane (T1-S1) plumb line with the supine CT scan for the entire cohort.

### The effect of shifting the location of the Instantaneous Centre of Rotation (ICR)

As just stated, we assumed that the coronal plane ICR was located at the centroid of a coronal plane projection of the intervertebral disc in question. Since there is uncertainty regarding the position of the ICR in the scoliotic spine, a sensitivity analysis was carried out to assess the effect of changing the ICR location on the estimated joint moments. In this sensitivity analysis, the assumed ICR location was shifted laterally by 10 mm in each direction (towards and away from) the convexity of the scoliotic curve.

## Results

The patient demographics and clinical data for each of the 20 AIS patients are presented in Table 1. The mean age of the group was 15.3 years (SD 2.3; range 11.9–22.3 years). All curves were right-sided major thoracic Lenke Type 1 with 11 patients further classified as lumbar spine modifier A, 4 as lumbar modifier B and 5 as lumbar modifier C. The mean thoracic Cobb angle was 52^°^ (SD 5.9; range 42–63^°^). The mean mass was 57.5 kg (SD 11.5; range 41–84.7 kg). Five patients were Risser grade 0, one patient was Risser grade 3, five patients were Risser grade 4 and nine patients were Risser grade 5.

**Table 1:** Demographics and clinical data (from standing radiographs) for the patients grouped by maximum apical coronal plane joint moment (JM)

JM Group	Pt ID	Age (yrs)	Patient Mass (kg)	Height (cm)	BMI (kg/m^2^)	Risser (0–5)	Major Cobb Angle (°)	Lumbar compensatory Cobb Angle (°)	Lenke class	Apex location	Max JM (Nm)
2.1–3 Nm	10	16.6	46.3	163	17.4	5	49	45	1A	T8/T9	2.9
	14	13.7	83	163	31.2	4	58	53	1C	T8	3
Mean	-	15.2	64.7	163	24.3	-	54	49	-	-	2.9
3.1–4 Nm	5	14.5	59.6	167	21.4	5	45	19	1A	T8/T9	3.1
	11	15.4	59.6	161	23	5	44	21	1A	T8	3.2
	18	16.5	54.6	164	20.3	4	50	40	1A	T8/9	3.3
	8	14.7	45.5	156	18.7	5	47	20	1A	T10	3.4
	17	16.7	54	167	19.4	3	50	24	1A	T7/8	3.5
	19	11.9	54.7	146	25.7	0	52	38	1B	T9/10	3.5
	12	13.8	55.4	171	18.9	4	54	38	1C	T7	3.7
	7	13	47	160	18.4	0	63	28	1A	T9	3.8
	3	16.2	63	165	23.1	5	42	37	1C	T7/8	3.9
Mean	-	14.7	54.8	162	21	-	50	29	-	-	3.5
4.1–5 Nm	2	15.2	58.9	173	19.7	5	45	42	1C	T8	4.3
	20	15.1	48.7	170	16.9	0	50	25	1A	T8/T9	4.3
	9	14.8	64	165	23.5	5	48	38	1B	T9/T10	4.3
	6	14	49	164	18.2	0	62	35	1A	T9	4.4
	16	14.7	58	160	22.7	4	55	35	1B	T8/9	4.6
Mean	-	14.7	56	166	20.2	-	52	35	-	-	4.4
5.1–6 Nm	15	19.2	52.5	168	18.6	5	58	38	1A	T8	5.2
	1	12.3	41	157	16.6	0	54	23	1A	T8/9	5.4
	4	22.3	70.8	175	23.1	5	48	40	1C	T8/9	5.5
Mean	-	17.9	54.8	167	19.5	-	53	34	-	-	5.4
6.1–7 Nm	13	14.8	84.7	161	32.7	4	57	40	1B	T7	7.1
Group Mean	-	15.3	57.5	163.8	21.5	3	52	34	-	-	4.1

The mean data of each JM group is shown in bold

**Fig. 3:**
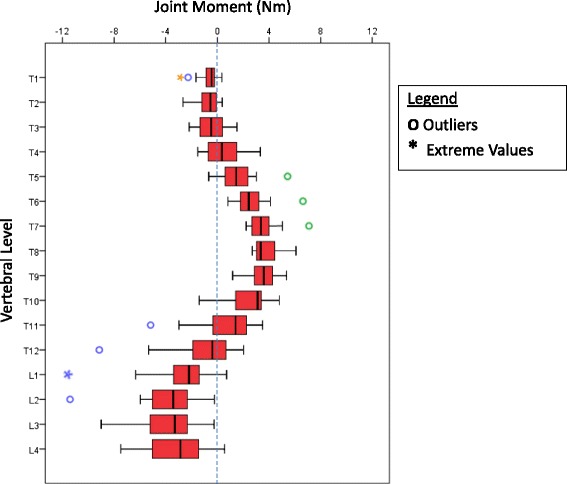
Coronal plane joint moments for the 20 patients. The circles represent outliers (those data points between 1.5 and 3.0 times the interquartile range outside the first and third quartiles respectively) and the asterisks represent extreme outliers (more than 3.0 times the interquartile range outside the first/third respectively). The scale shows joint moments in Nm and + ve is a clockwise moment. Patient 5 is shown in orange, Patient 13 is shown in green and Patient 14 is shown in blue

**Fig. 4:**
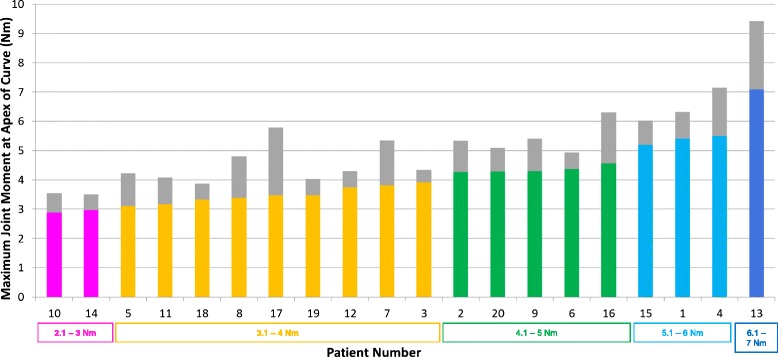
Coronal plane joint moment (Nm) acting at the apex of the curve for the 20 patients. The grey bars are the estimated increase in the joint moment according to the increase in Cobb angle that occurs in standing relative to the supine position

Figure 3 shows a boxplot of joint moment vs vertebral level for the entire cohort. The joint moment distributions for each of the 20 patients in the study are given individually in the Additional file 1. As expected, the maximum joint moment for the major curve in each patient occurred at the joint closest to the apex of the curve, and the magnitudes of these peak moments for the entire patient group are compared in Fig. 4. It should be noted that there were in some patients, large joint moments in the lumbar spine. These larger moments were due in part to the cumulative weight of the body segments above the lumbar joints. The lumbar moments showed greater variability than those for the thoracic region, most probably due to the variability in the presence of a large compensatory lumbar curve.

**Fig. 5:**
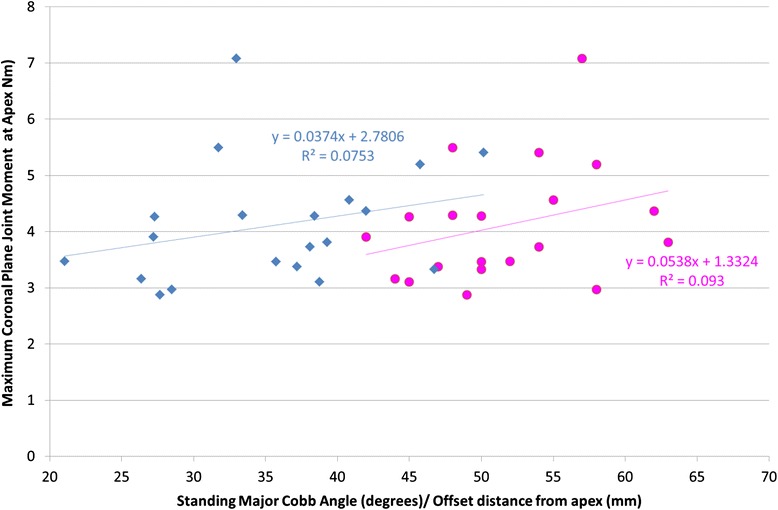
Scatter plot of coronal plane joint moment at the apical joint versus (i) clinically measured major cobb angle (blue dots) and (ii) lateral deviation of the ICR of the apical intervertebral joint (pink dots). Neither of the regressions are statistically significant, however when the outlier (patient 13 = 7.1 Nm moment) is removed from the regression, the correlation between apical joint moment and apical lateral deviation is near-significant (*P* < 0.10)

A scatter plot of the maximum apical joint moment versus clinical major Cobb angle is shown in Fig. 5. Regression analysis found no statistically significant relationship between maximum joint moment at the apex and clinically measured major Cobb angle (*P* = 0.192). This plot also shows the maximum apical joint moment plotted against the horizontal offset distance between the apical disc ICR and the straight line joining the T1/T2 and L4/L5 disc ICRs. Again, no statistically significant relationship was found between maximum joint moment at the apex and the offset distance (*P* = 0.280). However, upon removal of Patient 13 (outlier patient of mass 84 kg with an apical joint moment of 7 Nm), we found a *p*-value close to statistical significance (*p* = 0.079) for the relationship between apex horizontal offset and apical joint moment, but not for Cobb (*P* = 0.364).

**Fig. 6:**
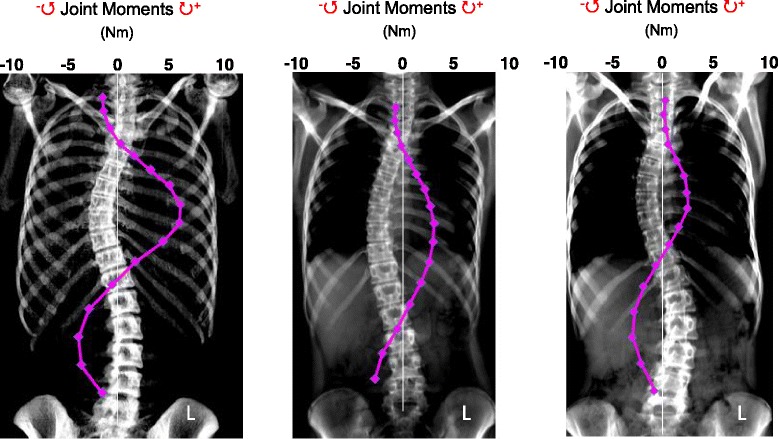
Examples of coronal CT images (from Patients 4, 8 and 10) showing the variability of joint moment distribution for three patients (Anterior-Posterior view). The scale shows joint moments in Nm and + ve is a clockwise moment

Visual inspection of the joint moment distributions for each patient shows significant variability according to spinal curve shape. This is highlighted in Fig. 6, where three patients from the study (Patients 8, 12 and 19) all having similar major Cobb angles (47, 54 and 52° with peak joint moments of 1.86, 2.29 and 3.06 Nm respectively) are compared.

Multi-linear regression found no statistically significant relationship between joint moment distribution and four independent variables: patient mass (*p* = 0.50), age (*p* = 0.35), Risser sign (*p* = 0.10) and Lenke modifier (*p* = 0.78) with an R-squared value 18 % (for all four variables combined).

Because the foregoing analysis was performed on supine CT anatomy, we also include an estimated correction for supine to standing change in Cobb angle (grey bars in Fig. 4). This correction was performed by measuring the Cobb angle on both the supine CT image and the clinical standing X-ray as described in a previous study [23]. The difference between the two Cobb angle measures was divided by the patient’s supine Cobb angle, and used to scale the joint moment at the apex to provide an estimate of the joint moment in standing. In this way, the estimated increase in joint moment varies by patient depending on the flexibility of the spine. Shifting the ICR by 10 mm towards or away from the convexity of the spine, changed the joint moment at that level by a mean of 9.0 %, showing that calculated joint moments were relatively insensitive to the assumed ICR location.

With regard to patient positioning on the scanner bed, of the 20 patients analysed, only three of the plumb lines (Patients 2, 16 and 17) differed by 2 cm or more between standing radiograph and supine CT.

### Intra-observer variability

Intra-observer variability for the torso segment thickness, mass and slice location has previously been reported [18]. With regard to the sensitivity of the ICR location, the abovementioned 9 % change in response to a 10 mm shift towards or away from the curve convexity suggests that a relative shift of the ICR by 2–3 mm (a typical value for intra-observer variability in ICR location selection) would have a negligible (< 3 %) effect on the resulting coronal plane joint moment.

## Discussion

Previous studies have suggested that gravity plays a key role in driving deformity progression in AIS. The primary aim of this study, therefore, was to take advantage of an existing 3D CT dataset to estimate joint moments in the standing scoliotic spine; to assess the magnitude and distribution of coronal plane joint moments occurring in AIS patients with moderate deformities; and to assess whether there is a relationship between joint moment magnitude and curve severity.

The results from this study have shown that there is a consistent pattern of joint moments in this group of patients with the same type of deformity (i.e. right-sided Lenke Type 1, thoracic curves). Despite this, the maximum joint moment in the major curve always occurred at the apex of the thoracic curve, although some patients also displayed large joint moments in the lumbar compensatory curve.

When dividing the patient cohort into subgroups of patients with similar joint moments, no clear trends were observed with existing clinical measures (as shown in Table 1). Intuitively one would expect apical joint moments to increase with Cobb angle. However Cobb angle is a limited measure from a biomechanical perspective because two scoliotic curves can have the same Cobb angle but differ widely in the apical moment due to differences in the offset of the apical vertebra. This is the reason that we included a scatter plot of apical joint moment vs apical offset in Fig. 5, where we found that whilst Cobb versus apical joint moment was not statistically significant, differences in offset versus apical moment was close to statistical significance. Similarly, there was no clear relationship between patient age, mass, Lenke modifier and Risser sign with joint moment distribution, although it is worth noting that the heaviest patient in the study also had the highest apical joint moment by a substantial margin.

Because the CT scans were performed in the supine position and Cobb angle magnitudes in this position are known to be 7–10° smaller than those measured in standing [23, 24], the joint moments in actual standing (as opposed to the simulated standing analysis performed here) would be expected to be greater than those calculated. The effect of the supine vs standing position on joint moments was estimated in Fig. 4, with the apical joint moment increasing by an average of 1.08 Nm (range 0.43–2.34 Nm).

It is also important to consider patient alignment on the scanner bed because the gravity vectors were always assumed to act along the scanner bed coordinate system z-axis in this analysis. We note that only three of the patients had a difference in plumb line of more than 2 cm between standing clinical radiograph and supine CT scan, suggesting that perhaps these three patients were not ideally aligned for their supine scan. Two of these patients (16 and 17) do exhibit relatively high lumbar spine joint moments, which may be an artefact of their positioning on the scanner bed. The other patient (Patient 2) does not exhibit high lumbar joint moments, but this could be either due to the fact that they were positioned ‘straight’ on the scanner bed when in fact they have an appreciable lateral offset when standing, or that they were positioned slightly angled on the scanner bed in the opposite direction to Patients 16 and 17, thus cancelling rather than exacerbating the calculated lumbar moment. Thus a potential improvement to our methodology in future could be attempting to standardise patient positioning on the scanner bed, although we note that this standardisation would have to be careful not to ‘correct’ a coronal plane plumb line offset which is actually present in the patient.

The technique presented in the current study was performed by a single observer for research purposes and hence inter-observer variability in a clinical setting was not assessed. The authors therefore do not anticipate that the findings of the current study should be used to influence clinical decision making until further validation studies of this technique are performed.

Whilst the present study does not include any muscle loading, we believe that the use of static analysis to calculate the gravity-induced joint moments was an appropriate starting point; particularly for analysis of loading in the thoracic spine. Firstly, the rib cage has been shown to significantly increase stability of the thoracic spine (by 40 % in flexion/extension, 35 % in lateral bending and 31 % in axial rotation [25]). Secondly, lateral bending tests on cadaveric lumbar spine motion segments have shown that applied moments of 4.7–10.6 Nm of lateral bending result in rotations of 3.51–5.64° [26, 27]. Taken together, these studies suggest that the coronal plane moments (up to 7 Nm) estimated here, could be resisted by passive osseoligamentous structures undergoing a few degrees of lateral wedging. The extent to which muscle activation is involved in resisting coronal plane moments in standing AIS patients is unclear. Reports in the literature regarding muscle activation patterns in AIS are limited, particularly for the thoracic spine and in the standing position. Whilst Finite Element models have been developed to assess muscle asymmetry, the focus is primarily only on the lumbar spine [10, 28], which is inherently different to the thoracic spine (due to the absence of the ribcage).

In future, deformity progression could be assessed using sequential imaging techniques (as opposed to a single scan taken at one instant in time). Comparing the moments estimated at a particular time point to subsequent progression of a patient’s curvature could provide valuable information on whether there is a threshold beyond which the joint moments are large enough to drive the deformity. It will also be important to extend the coronal plane analysis performed here to three dimensions, to allow a full accounting of the effect of coronal, sagittal and transverse plane moments and forces on deformity progression [29]. Such biomechanical understanding would provide useful insights into the effectiveness of bracing and other treatment strategies in individual patients.

## Conclusions

Torso segment masses were used to estimate joint moments in the thoracic and lumbar spines of scoliosis patients. This study suggests that significant gravity-induced coronal plane joint moments act on the spines of scoliosis patients. Coronal plane joint moments of up to 7 Nm are present at the apical level of the major curve, increasing to an estimated 9 Nm in the upright position. There is substantial variation in joint moment distributions between patients with apparently similar curve type and magnitude. Although the relationship between magnitude of moment and deformity severity in individual patients remains unclear, gravity is a potential driving factor in coronal plane scoliosis progression, which may help to explain the mechanics of AIS. In terms of clinical implications for deformity correction, this study suggests that quite large forces for both internal instrumentation and external bracing are required to produce moments capable of countering those induced by gravity, and further (three dimensional) development of the approach used here may provide a quantitative foundation for treatments aimed at halting and correcting deformity progression.

## Additional file

Additional file 1:
**Individual Coronal Plane Joint Moment Plots (Anterior-Posterior views on reformatted CT image).** The scale shows joint moments in Nm and + ve is a clockwise moment. (DOCX 2095 kb)

## Footnotes

1 ‘Segmental mass’ here refers to the mass of an imaginary slice through the torso at the level of the vertebra in question. The thickness of the slice is shown in Fig. 1(c).

2 In Engineering Mechanics a moment is a twisting torque due to a force acting at a distance from a centre of rotation. Since the force is due to gravity acting on segments of body mass and the centres of rotation are spinal joints we refer to these moments as gravity-induced joint moments.
