# Supplementary material for: Gravity-induced coronal plane joint moments in adolescent idiopathic scoliosis
Source: Scoliosis. 2015 Dec 17;10:35. doi: 10.1186/s13013-015-0060-9 (PMC4682283; doi:10.1186/s13013-015-0060-9)
Supplement: Additional file 1: — Individual Coronal Plane Joint Moment Plots (Anterior-Posterior views on reformatted CT image). The scale shows joint moments in Nm and + ve is a clockwise moment. (DOCX 2095 kb) [file 13013_2015_60_MOESM1_ESM.docx]

^-^⭯ Joint Moments ⭮^+^

^(Nm)^

**^-10 -5 0 5 10^**

^-^⭯ Joint Moments ⭮^+^

^(Nm)^

**^-10 -5 0 5 10^**

^-^⭯ Joint Moments ⭮^+^

^(Nm)^

**^-10 -5 0 5 10^**

^-^⭯ Joint Moments ⭮^+^

^(Nm)^

**^-10 -5 0 5 10^**

^-^⭯ Joint Moments ⭮^+^

^(Nm)^

**^-10 -5 0 5 10^**

^-^⭯ Joint Moments ⭮^+^

^(Nm)^

**^-10 -5 0 5 10^**

^-^⭯ Joint Moments ⭮^+^

^(Nm)^

**^-10 -5 0 5 10^**

^-^⭯ Joint Moments ⭮^+^

^(Nm)^

**^-10 -5 0 5 10^**

^-^⭯ Joint Moments ⭮^+^

^(Nm)^

**^-10 -5 0 5 10^**

^-^⭯ Joint Moments ⭮^+^

^(Nm)^

**^-10 -5 0 5 10^**


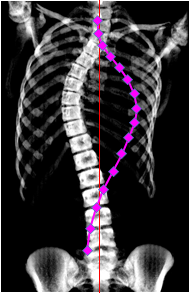

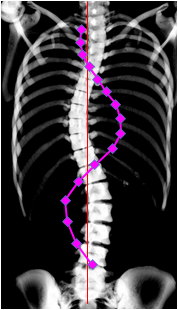

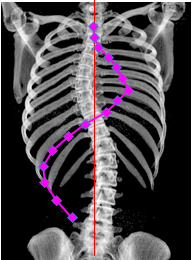

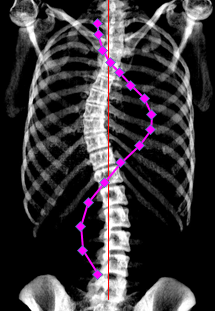

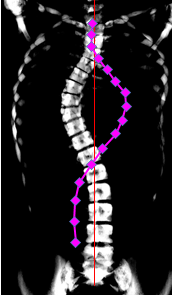

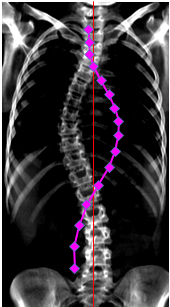

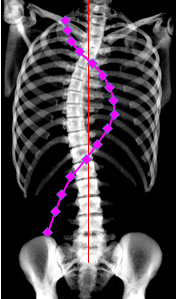

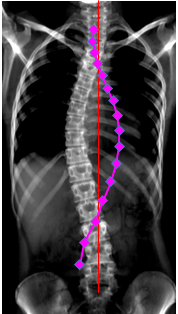

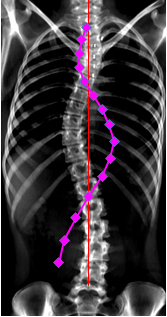

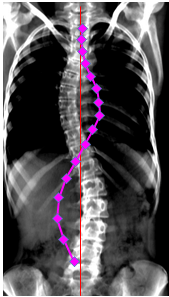


**Patient 1**

Cobb angle 54°

**Patient 3**

Cobb angle 42°

**Patient 4**

Cobb angle 48°

**Patient 2**

Cobb angle 45°

**Patient 5**

Cobb angle 45°

**Patient 6**

Cobb angle 62°

**Patient 7**

Cobb angle 63°

**Patient 8**

Cobb angle 47°

**Patient 9**

Cobb angle 48°

**Patient 10**

Cobb angle 49°


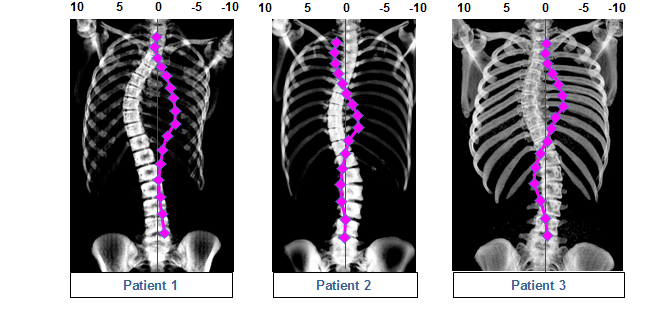


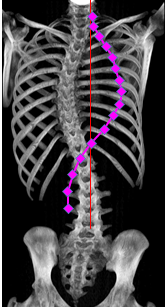

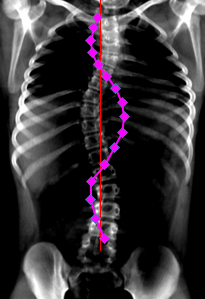

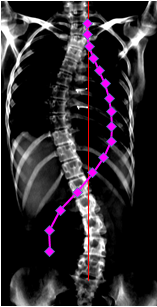

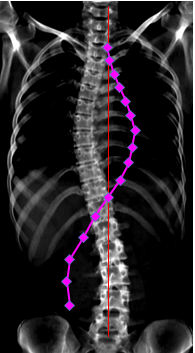

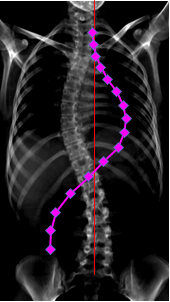

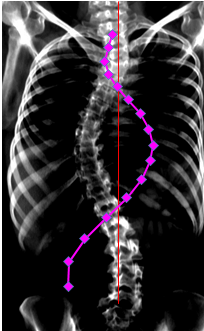

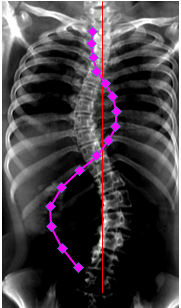

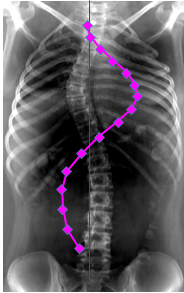

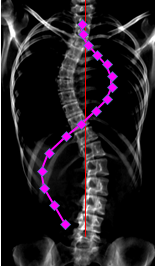

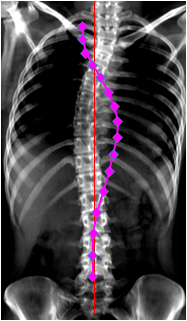


**Patient 15**

Cobb angle 58°

^-^⭯ Joint Moments ⭮^+^

^(Nm)^

**^-10 -5 0 5 10^**

**Patient 19**

Cobb angle 52°

^-^⭯ Joint Moments ⭮^+^

^(Nm)^

**^-10 -5 0 5 10^**

**Patient 18**

Cobb angle 50°

^-^⭯ Joint Moments ⭮^+^

^(Nm)^

**^-10 -5 0 5 10^**

**Patient 17**

Cobb angle 50°

^-^⭯ Joint Moments ⭮^+^

^(Nm)^

**^-10 -5 0 5 10^**

^-^⭯ Joint Moments ⭮^+^

^(Nm)^

**^-10 -5 0 5 10^**

^-^⭯ Joint Moments ⭮^+^

^(Nm)^

**^-10 -5 0 5 10^**

^-^⭯ Joint Moments ⭮^+^

^(Nm)^

**^-10 -5 0 5 10^**

**Patient 14**

Cobb angle 58°

**Patient 13**

Cobb angle 57°

^-^⭯ Joint Moments ⭮^+^

^(Nm)^

**^-10 -5 0 5 10^**

^-^⭯ Joint Moments ⭮^+^

^(Nm)^

**^-10 -5 0 5 10^**

**Patient 12**

Cobb angle 54°

^-^⭯ Joint Moments ⭮^+^

^(Nm)^

**^-10 -5 0 5 10^**

**Patient 11**

Cobb angle 44°

**Patient 16**

Cobb angle 55°

**Patient 20**

Cobb angle 50°

^+^⭯ Joint Moments ⭮^-^

^(Nm)^

^+^⭯ Joint Moments ⭮^-^

^(Nm)^

^+^⭯ Joint Moments ⭮^-^

^(Nm)^

^+^⭯ Joint Moments ⭮^-^

^(Nm)^

^+^⭯ Joint Moments ⭮^-^

^(Nm)^
